# Spousal Activity Limitations and Depressive Symptoms: Benefits of Spousal Caregiving and Costs of Spousal Pain

**DOI:** 10.1093/geroni/igaa057.1138

**Published:** 2020-12-16

**Authors:** Sae Hwang Han, Kyungmin Kim, Jeffrey Burr

**Affiliations:** 1 University of Texas at Austin, Austin, Texas, United States; 2 University of Massachusetts Boston, Boston, Massachusetts, United States

## Abstract

Experiencing difficulties in performing basic activities of daily living poses significant challenges for older adults living with such limitations and also for their spouses. A growing body of evidence demonstrates cross-spousal linkages between activity limitations and depressive symptoms. However, under what conditions these linkages may be strengthened or weakened has received little attention in the literature. We addressed this gap by examining whether a) providing spousal caregiving and b) spousal pain moderated the link between spousal activity limitations and one’s own depressive symptoms. We used seven waves of longitudinal household data from the Health and Retirement Study (2004-2016; N=12,369) to estimate within-person associations between spousal activity limitations and depressive symptoms, focusing on the moderating roles of caregiving behavior and spousal pain. In particular, asymmetric fixed effects models were used to estimate the unique effects of transitioning into a spousal caregiver role in the context of spousal activity limitations. Results from multilevel models were gendered. For wives (but not for husbands), transitioning into a caregiver role to provide spousal care alleviated depressive symptoms associated with spousal activity limitations, whereas depressive symptoms were increased when husbands with activity limitations also reported frequent, moderate to severe pain. Our findings indicate that the link between spousal activity limitations and depressive symptoms is not uniform, and that the cross-spousal association may best be understood when relevant contextual factors are considered. The findings are also in line with recent studies showing that caregiving may also lead to enhanced well-being and reduced mortality risk under some circumstances.

